# Case Report: Severe Rhabdomyolysis and Multiorgan Failure After ChAdOx1 nCoV-19 Vaccination

**DOI:** 10.3389/fimmu.2022.845496

**Published:** 2022-03-17

**Authors:** Emilia Cirillo, Ciro Esposito, Giuliana Giardino, Gaetano Azan, Simona Fecarotta, Stefania Pittaluga, Lucia Ruggiero, Ferdinando Barretta, Giulia Frisso, Luigi Daniele Notarangelo, Claudio Pignata

**Affiliations:** ^1^Departments of Translational Medical Sciences, Pediatric Section, Federico II University of Naples, Naples, Italy; ^2^Department of Transplants, A. Cardarelli Hospital, Naples, Italy; ^3^Laboratory of Pathology Center for Cancer Research, National Cancer Institute, National Institutes of Health, Bethesda, MD, United States; ^4^Department of Neuroscience, Reproductive and Odontostomatological Science, Federico II University of Naples, Naples, Italy; ^5^Department of Molecular Medicine and Medical Biotechnology , Federico II University of Naples, Naples, Italy; ^6^Laboratory of Clinical Immunology and Microbiology, National Institute of Allergy and Infectious Diseases, National Institutes of Health, Bethesda, MD, United States

**Keywords:** vaccination, cytokine storm, anakinra, eculizumab, rhabdomyolysis, case report

## Abstract

**Background:**

Severe skeletal muscle damage has been recently reported in patients with SARS-CoV-2 infection and as a rare vaccination complication.

**Case summary:**

On Apr 28, 2021 a 68-year-old man who was previously healthy presented with an extremely severe rhabdomyolysis that occurred nine days following the first dose of SARS-CoV-2 ChAdOx1 nCov-19 vaccination. He had no risk factors, and denied any further assumption of drugs except for fermented red rice, and berberine supplement. The clinical scenario was complicated by a multi organ failure involving bone marrow, liver, lung, and kidney. For the rapid increase of the inflammatory markers, a cytokine storm was suspected and multi-target biologic immunosuppressive therapy was started, consisting of steroids, anakinra, and eculizumab, which was initially successful resulting in close to normal values of creatine phosphokinase after 17 days of treatment. Unfortunately, 48 days after the vaccination an accelerated phase of deterioration, characterized by severe multi-lineage cytopenia, untreatable hypotensive shock, hypoglycemia, and dramatic increase of procalcitonin (PCT), led to patient death.

**Conclusion:**

Physicians should be aware that severe and fatal rhabdomyolysis may occur after SARS-CoV2 vaccine administration.

## Introduction

Severe skeletal muscle damage has been recently reported in patients with SARS-CoV-2 infection. In these subjects, weakness due to severe myopathy can adversely affect the outcome in SARS-CoV-2 pneumonia. Despite this, the mechanism of skeletal muscle damage remained elusive (1). A cytokine-mediated direct muscle cell damage has been taken in consideration as the main cause of this condition since rhabdomyolysis has been reported after several days (2-4 weeks) of COVID-19 symptoms onset and coincided with the peak of inflammatory markers (2, 3). In support of this hypothesis, viral particles were identified in skeletal muscle-infiltrating macrophages in patients who developed rhabdomyolysis following infection with the related Middle East Respiratory Syndrome (MERS) virus (4). In the last months, progress has been made with the development and authorization of vaccines and antibody therapies. Vaccine strategies are directed at the viral spike protein (5). At the date of June 24, a total of 2.624.733.776 vaccine doses have been globally administered of which 48.316.875 doses in Italy. Although the beneficial effects of vaccines outweigh vaccination associated risks, life-threatening thrombosis and thrombocytopenia has been observed in a few individuals who received recombinant adenoviral vector encoding spike protein of SARS-CoV-2 ChAdOx1 nCov-19 (AstraZeneca), probably due to nCov-19 vaccine-induced platelet-activating antibodies against platelet factor 4 (PF4) (6, 7). Since its approval in Italy on January 29, 2021, cerebral venous thrombosis and/or venous thrombosis in atypical site (e.g.: thrombosis of the splanchnic veins) have been reported by the Regulatory Italian Agency (Agenzia Italiana del Farmaco, AIFA, - COVID-19 Vaccine Surveillance Report), with a frequency of approximately 1 event per 100,000 doses administered (8, 9). These events occurred after the administration of the first dose of ChAdOx1 nCov-19 vaccine and mostly under the age of 65. Further systemic and more common adverse events usually are self-limiting and include headache, fatigue, diarrhea, fever, arthralgia and myalgia.

Rhabdomyolysis has been reported as a rare vaccination complication after influenza A H1N1 vaccine, and recombinant zoster immunization (10). Furthermore, a severe form of rhabdomyolysis after a COVID-19 AstraZeneca vaccine has been reported in a young patient who also had a carnitine palmitoyltransferase II (CPT II) deficiency (11), in a 21-year-old male with a history of asthma after the first dose of the Pfizer/BioNTech COVID-19 vaccine and more recently in a patient who also developed anti-neutrophil cytoplasmic antibody (ANCA)-associated vasculitis (AAV) and pauci-immune crescentic glomerulonephritis after two doses of Pfizer-BioNTech COVID-19 mRNA vaccination (12, 13). These subjects recovered after appropriate therapies. Here we report on a 68-year-old previously healthy man who developed a severe and fatal rhabdomyolysis with multi-organ failure including liver, kidney and lung, 9 days after ChAdOx1 nCov-19 vaccine administration. A successful response to multi-target biologic immunosuppressive therapy was achieved after 36 hours. However, unfortunately, the patient died at day + 48 from vaccination due to an accelerated phase, characterized by untreatable sudden hypotension, severe hypoglycemia, bone marrow failure associated to a remarkable increase of procalcitonin (PCT).

## Case Report

**Figure 1:**
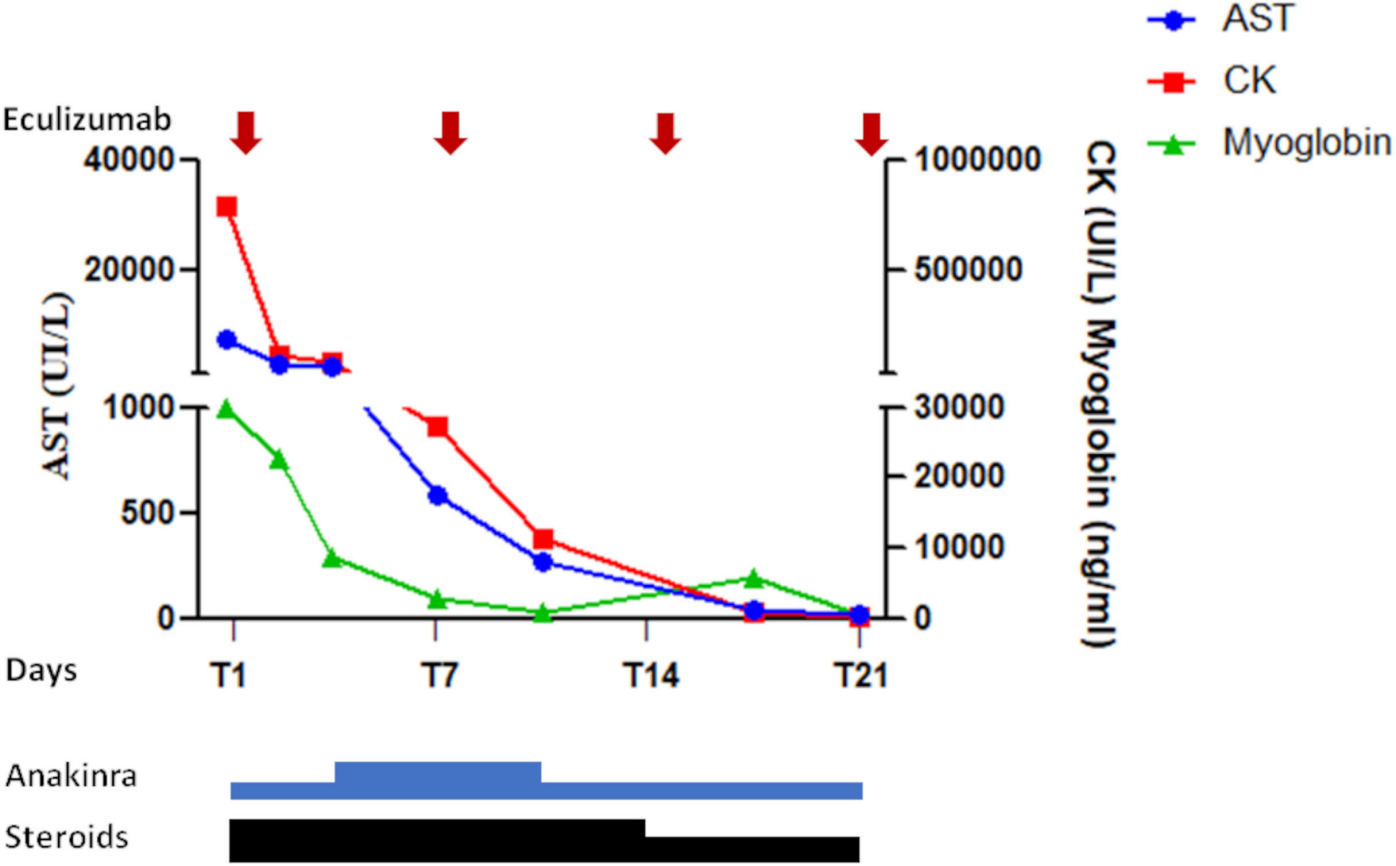
*Response to the multi-targeted treatment in the first 21 days*: A significant decrease of creatine kinase, myoglobin, and AST was achieved since the first 36 hours from the treatment. A further progressive reduction was observed in the following days. Due to severe acute kidney injury anakinra was administered at the dose of 200 mg/die in the first 3 days. The same dose was also administered from day+ 11. From day +4 to day +10 it was administered at the dose of 300 mg/die. From day +1 to 14 the patient also received methylprednisolone (100 mg/die), subsequently de-escalated to 60 mg/die. Vertical arrows indicate the four doses of eculizumab.

The patient was a previously healthy 68-year-old man, with no risk factors, who received the first dose of ChAdOx1 nCov-19 vaccine on April 19, 2021. He denied any further assumption of drugs except for fermented red rice and berberine supplement. Blood testing was performed 15 days before vaccination since the patient worried about adverse events related to the procedure and it was found normal. After 9 days, he was admitted at the emergency room because of acute malaise, dispnoea, severe abdominal pain, myalgia and difficulty with ambulation. Patient’s vital signs at the admission at the ICU were as follows: pressure 156/91 mmHg, hearth rate 97 beats/min, respiratory rate 14 breaths/min. His temperature was 36.2°C and he always remained apyretic, even though the patient was under steroid treatment. A good pressure control was obtained after 48 hours from the admission. A severe interstitial pneumopathy was documented at the computed tomography (CT) scan. A progressive contraction of diuresis was also reported. At clinical evaluation, the patient showed a slight clouding of the sensory, associated with weakness in the four limbs without any other focal neurological deficits. Several atrial fibrillation events were successfully treated with beta-blockers. Laboratory tests performed in the first hours from symptom onset showed elevated serum levels of alanine aminotransferase (ALT): 1432 U/L, aspartate aminotransferase (AST): 7815 U/L, creatine phosphokinase (CK): 376 UI/L, creatinine: 3.72 mg/dl, blood urea nitrogen (BUN): 89 mg/dl, lactic acid: 4.2 meq/L, as well as hyperkalemia (7.3 mg/dl), hypocalcemia (5.8 mg/dl) and lymphopenia (380 cells per mm^3^) (**Table 1**). Inflammatory markers were elevated, and a rapid and dramatic increase of CK and of myoglobin was observed, as illustrated in detail in the Table. Multiple trans-thoracic echocardiography evaluations and serum troponin levels were normal except a slight increase of troponin I on day +1 after the admission (+10 from the vaccination). IgG levels were 638 mg/dl (normal values 700-1600 mg/dl), IgA levels 179 mg/dl (normal values 70-400 mg/dl), and IgM 59 mg/dl (normal values 40-230 mg/dl). Because of the rapid progression of multi-organ failure, the patient was transferred to intensive care unit (ICU). Echo-color doppler examination excluded a deep vein thrombosis (DVT) in the explored vessels. Abdomen CT was normal. No infections were identified at blood, urine and bronchial cultures. Serological tests for hepatitis A, B or C, varicella–zoster virus, cytomegalovirus, rubella virus and *Toxoplasma gondii*, were negative. Polymerase chain reaction (PCR) nasal swab for SARS-CoV-2 was negative. Serum IgG or IgM antibodies to nucleocapsid and SARS-CoV-2 spike antigens were evaluated 10 and 14 days after vaccine administration by electro-chemiluminescence immunoassay (ECLIA), respectively, and were negative. Further common causes of rhabdomyolysis including trauma, strenuous exercise, recent surgery, alcohol use, toxic, autoimmune disorders and malignancies were excluded. Despite appropriate intravenous fluid therapy, a progression of the kidney injury was documented, requiring continuous renal replacement through venovenous hemodialysis (CRRT-CVVHD). On day +10 from the vaccination the patient was intubated due to severe respiratory failure (PaO_2_/FiO_2_: 140 mm/Hg) and respiratory muscle weakness. Since the rapid increase of the inflammatory markers and of the muscle injury were suggestive of a cytokine storm, intravenous high-dose methylprednisolone (100 mg daily) and subcutaneous anakinra, an interleukin-1–receptor antagonist, were started. Due to the severe acute phase, anakinra was administered at the dose of 200 mg daily during the first 3 days, 300 mg daily from day +4 to day +10, and 200 mg daily thereafter. Twenty four hours after the start of immunosuppressive therapy, eculizumab (600 mg per week) was added to block a potential complement mediated rhabdomyolysis. CK and AST levels progressively decreased (**Figure 1**) and after 17 days of treatment CK levels were close to normal, even though weakness of respiratory muscles persisted requiring tracheostomy. Despite a significant reduction of inflammatory markers, lymphopenia and kidney injury never recovered.

**Table 1:** Main laboratory features of the patient.

Clinical phase	Normal range		Acute				Improvement				Accelerated		
**Time from admission-days** (hours)^a^		-15	0	0 (+ 3)	+1 (+14)	+1 (+24)	+7	+14	+21	+28	+35	+35 (+6 h)	+36
**Time from vaccination (days)**		-5	+9	+9	+10	+10	+16	+23	+30	+38	+46	+46	+47
**White-cell count (per mm^3^)**	4000-10000	4600	17110	17800	14870	14370	12170	15320	7390	5330	2730	670	330
**Neutrophil count (per mm^3^)**	1500-7000	1850	15760	16260	13560	13160	11070	13810	6100	4620	2390	510	70
**Lymphocyte count (per mm^3^)**	1000-3700	2140	380	400	310	560	390	750	690	320	280	100	220
**Platelet count (per mm^3^)**	150000-400000	144000	226000	243000	179000	154000	122000	155000	42000	40000	30000	21000	28000
**Total hemoglobin (g/dl)**	13-17	13.9	16.3	16.5	14	13.9	9.2	8.1	7.0	7.3	8.9	8.8	9.5
**D-dimer (ng/ml)**	0-250	<0.2	NA	2627	NA	1479	2399	1906	525	328	173	169	148
**Lactate dehydrogenase (UI/L)**	135-225	NA	10690	NA	18354	NA	3191	726	514	305	NA	NA	NA
**C-reactive protein (mg/L)**	0-5	NA	141.12	NA	154.35	111.82	10.91	14	5.48	7.09	32.42	NA	124.83
**Interleukin 6 (pg/mL)**	0.1-7	NA	92.3	NA	NA	NA	10.3	19.7	NA	NA	NA	NA	NA
**Procalcitonin (ng/mL)**	0-0.5	NA	1.8	NA	NA	2.2	0.7	0.2	0.2	0.2	0.6	0.6	>100
**Fibrinogen (mg/dl)**	150-450	274	NA	682	NA	774	410	296	175	194	311	327	379
**Ferritin (ng/ml)**	30-400	NA	NA	NA	NA	715	1,048	521	498	NA	NA	NA	NA
**Creatinine (mg/dl)**	0.7-1.2	1.14	3.72	4.48	3.81	3.45	1.99	3.18	1.82	3.21	4.07	4.23	3.65
**Blood urea nitrogen (mg/dl)**	15-50	39	89	93	93	83	101	118	60	91	121	121	91
**Serum potassium (mEq/L)**	3.5-5.1	NA	7.3	7.7	6.6	6.6	7.1	7.0	8.2	8.3	8.0	7.3	6.8
**Serum calcium (mg/dl)**	8.6-10.2	NA	5.8	5.6	6.5	5.6	4.5	4.2	3.7	3.5	3.4	3.1	3.7
**Creatinine kinase (UI/L)**	0-190	NA	376	760960	652460	793280	20746	1881	490	202	559	545	495
**Myoglobin (ng/ml)**	25-72	NA	NA	1885	2204	30001	4441	704	594	1180	1404	1349	1723
**Aspartate aminotransferase (UI/L)**	0-40	20	7815	8764	7070	6899	583	62	29	22	37	34	42
**Alanine aminotransferase (UI/L)**	0-40	21	1432	1737	1440	1469	364	96	12	8	4	4	4
**Troponin I (ng/mL)**	0-0.3	NA	NA	0.3	1.02	1.61	0.1	0.12	0.11	0.1	0.21	0.27	0.9

a On day 0, 1 and 34 from the admission, multiple samples were collected. Acute phase, from 0 to +1 day; Improvement phase from +7 to +28 days, Accelerated phase from +35 to 36 days.

**Figure 2:**
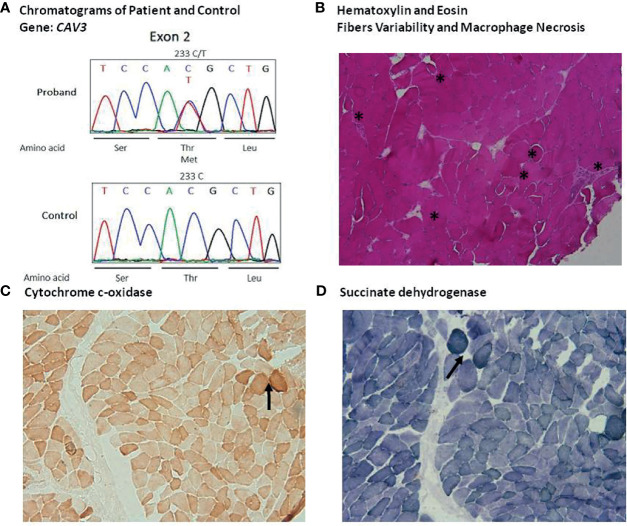
I*dentification of the T78M variant of CAV3 and quadriceps muscle histological finding***: (A)** Sequence chromatogram of the normal and patient genome. **(B)** Haematoxylin and eosin (HE) staining **(A)**, 10X), highlighting considerable variability of fibers size and morphology with several hypotrophic round fibers, degenerating fibers and macrophage necrosis (asterisks). Moreover, HE pointed out several slitting of fibers. **(C)** Cytochrome c-oxidase panel **(C)**, 10X) and **(D)** succinate dehydrogenase stain panel **(D)**, 10X) showing some fibers with modest alteration of oxidative metabolism (asterisks).

On day +14 form the vaccination (+ 5 from the admission), the patient underwent muscle biopsy. Tissue samples of quadriceps were analyzed at the Federico II facility (cryopreserved sample) and at the National Institutes of Health (formalin-fixed paraffin-embedded tissue) (protocol NCT04582903, “Send-in sample collection for comprehensive analyses of innate and adaptive immune responses during acute COVID-19 and convalescence”). Routine histological and immunohistochemical staining were performed. A myopathic process with focal necrotic myofibers and myophagocytosis was found (**Figure 2B**), along with a histiocytic infiltrate, with no CD20^+^ or CD3^+^ lymphoid cells. Only a modest alteration of oxidative metabolism was noted at the cytochrome c-oxidase (**Figure 2C**) and succinate dehydrogenase stain (**Figure 2D**). We next examined by Liquid Chromatography with tandem mass spectrometry (LC-MS/MS) the patient’ plasma acylcarnitine profile to rule out an inherited disorder of mitochondrial fatty acid β-oxidation, that could predispose to rhabdomyolysis under certain circumstances. A urine sample for urinary organic acids analysis was not available at that time. A slight increase of multiple serum acyl carnitines was found (C0, C2, C3, C4, C5, C5OH, C8, C3DC, C5DC, C12:1 C6 DC). Based on the hypothesis of a multiple acyl-CoA dehydrogenase deficiency (MADD), an exome sequence analysis targeted at 19 genes associated with mitochondrial fatty acid β-oxidation disorders was performed, and no genetic variations were detected. However, a heterozygous variant c.233C>T (p.Thr78Met), confirmed by Sanger sequencing, in caveolin 3 (*CAV3*) gene was found (**Figure 2A**). Furthermore, a c.800-1G>A in *MYH3* gene, encoding for Myosin heavy chain-embryonic (MyHC-emb) molecule, a skeletal muscle-specific contractile protein, was also documented. This variant, of unknown significance, is unlikely to have played a role in the clinical phenotype of our patient, since the gene is only expressed during embryonic and fetal stages of muscle development and its alteration is responsible for Freeman-Sheldon and Sheldon-Hall congenital contracture syndromes. After 32 days of treatment, for the persistence of lymphopenia (160 cells per mm^3^) and low platelet count, suggesting a bone marrow failure, anakinra was discontinued to rule out a potential drug induced effect. However, 5 days after discontinuation no recovery of bone marrow was observed and CK levels increased from 202 to 559 UI/L. A chest CT revealed a severe bilateral pleural effusion. *Pseudomonas aeruginosa* was isolated from bronchial aspirate and treated with meropenem. Worsening of clinical conditions, with multi-lineage cytopenia, hypoglycemia, dramatic increase of PCT, and severe hypotensive shock refractory to intravenous fluid replacement therapy led to death on day +48 from the vaccination, despite empiric antimicrobial therapy with liposomal amphotericin B, tigecycline, meropenem, fosfomycin, and cotrimoxazole.

## Discussion

Here, we report on a case of fatal rhabdomyolysis that occurred 9 days after vaccination for ChAdOx1 nCov-19, complicated by a multiorgan failure involving bone marrow, liver, kidney, and lung in a previously healthy subject. Multiorgan failure may be triggered by several infectious or other stimuli. Numerous infections can lead to rhabdomyolysis, including Mycoplasma pneumonia and Influenza A virus (14, 15). The proposed mechanisms for infection-induced rhabdomyolysis are tissue hypoxia, direct bacterial invasion of muscle, low oxidative and glycolytic enzyme activity, activation of lysosomal enzymes and mechanisms implicating endotoxins or autoimmunity due molecular mimicry. In particular, molecular mimicry may trigger the adaptive immune response, leading to the development of potentially pathogenic autoantibodies (16).

ChAdOx1 nCoV-19 is a SARS-CoV-2 vaccine comprising a replication-deficient simian adenovirus expressing full-length SARS-CoV-2 spike protein. Side effects associated to ChAdOx1 nCov-19 are usually mild and self-limiting. Venous thrombo-embolism in unusual sites with a fatal outcome has been occasionally reported (9). Following vaccinations, a few cases of rhabdomyiolysis in patients receiving influenza, varicella zoster and, more recently, also following mRNA vaccination, have been documented (10, 12). A severe rhabdomyolysis following COVID-19 AstraZeneca vaccine has been described in a patient with Carnitine palmitoyltransferase II (CPT II) deficiency, a disorder of long-chain fatty acid oxidation (FAO), caused by mutations in the CPT2 gene (11). We excluded a fatty acid β-oxidation disorder in our patient, and the slight increase of plasma acyl carnitines was interpreted as a consequence of a different dialytic removal of plasma acylcarnitines due to the severe acute kidney injury (17). Extended exome sequencing revealed a previously reported heterozygous c.233C>T (p.Thr78Met) variation in *CAV3* gene, encoding a protein expressed in muscles and myocardiocytes This variant, is located within the central hydrophobic transmembrane domain (amino acids 75106), and is predicted to be damaging according to *in-silico* tools. *CAV3* participates to structural assembly of caveolae, regulates ion channels, signaling, energy metabolism and mitochondria function (18). *CAV3* also participates in the development and repair of smooth, skeletal and cardiac muscles (19). Autosomal dominant *CAV3* variants have been associated with a group of inherited myopathies that include Rippling muscle syndrome, distal myopathy and familial hypertrophic cardiomyopathy (20). *CAV3* variants have also been reported in patients with asymptomatic hyperCKemia and long QT syndrome (18, 21). Although the pathogenetic role of the T78M variation is questionable, being also found in healthy population, and in particular among Southern Italians, it has been demonstrated that T78M CAV3 variant alters conformation or function of the ion channel, ultimately affecting membrane excitability (22, 23). In our patient, several episodes of easy-to-treat supraventricular arrhythmias were reported during the hospitalization. Even though we cannot exclude the role of this variant in the arrhythmic events observed, it should be mentioned that both the rapid accumulation of potassium in the serum and the hypocalcemia may have had a direct role in the myocardial phenotype.

It may be hypothesized that ChAdOx1 nCoV-19 vaccination has triggered the fatal rhabdomyolysis in a subject in whom the CAV3 T78M variant may have represented a risk factor for membrane fragility and decreased muscle fiber integrity. Of note, caveolin-dependent endocytic pathway also provides an entry route for several pathogenic viruses (24).

It is possible that in our patient immune-mediated muscle damage may have led to inflammation and necrosis of the muscles. In favor of such hypothesis there is the prompt dramatic positive response to the combined biologic therapy with anakinra and eculizumab, that block both the initial and the efferent complement-mediated phase of the immune response. Moreover, IL-6 levels were elevated. Unfortunately, no data are available on serum levels of IL-1 and other pro-inflammatory mediators. Anakinra has been proven successful in several hyper-inflammatory circumstances, such as macrophage activation syndrome, haemophagocytic lymphohistiocytosis (sHLH) or severe COVID-19 pneumonia (25–27). Bone marrow examination was not performed in our patient. Hemophagocytyc lymphohistocytosis was excluded since the only clinical criteria was high serum levels of ferritin and the reactive hemophagocytic syndrome diagnostic score (HScore), used to quantify likelihood of a hemophagocytic syndrome, was not suggestive of the syndrome (HScore range: 43-53, probability <1%) (28). Furthermore, the extended exome sequencing excluded primary fHLH and other inborn errors of immune system. Recently it has been reported a role of complement in the pathophysiology of COVID-19. Several cases of complement activation have also been reported in patients with paroxysmal nocturnal hemoglobinuria after SARS-Cov-2 vaccine. In these cases, SARS-CoV-2 vaccine seems to mainly act through the activation of classical pathway (29). Even though complement was not tested and abnormalities could not be excluded at that time, eculizumab, a C5 inhibitor, was administered. Furthermore, it has been suggested that a blockade of C5a may be crucial for inhibition of the cytokine storm (30, 31). Up to date, none of the viruses implicated as possible triggers of myositis have been shown to directly infect muscle fibers. However, in mice model, it has been documented that liver and spleen macrophages, sequester large quantities of adenovirus derived (AD) vectors after their intravenous administration (32, 33). Of note, in damaged muscles an abundant expression of fiber-dependent receptor for coxsackie virus and adenovirus, has been reported (34). Furthermore, viral particles in macrophages infiltrating skeletal muscles have been found in MERS-CoV patients (4).Viruses induce a viral specific immune T cell-mediated response, or, as observed in our patient, a macrophage-mediated response, eventually leading to muscle fiber infiltration and exuberant pro-inflammatory cytokines production. In our case, the vaccine consisted of a defective virus. AD vectors are currently used worldwide in gene therapy clinical trials. The experience gained with the use of these modified viruses has highlighted the possibility that adenoviral capsid proteins, may induce a rapid activation of innate immune system and cytokine storm, that result in multi organ failure (35). Indeed, elevated serum levels of IL-6 and IL-1 and activation of the complement classical pathway have been reported in humans after injection of high numbers of AD vector particles (33). A biopsy of the quadriceps muscle, performed four days after the initiation of immunosuppressive therapy, revealed fiber necrosis with phagocytosis and influx of histiocytes, associated with a significant increase of the vascular component. Lack of a lymphocytic infiltrate in the biopsy may be explained by the immunosuppression and by the severe lymphopenia that was present from the time of onset of clinical symptoms. In this regard, it should be noted that lymphopenia has not been previously reported in recipients of the ChAdOx1 nCoV-19 vaccine.

In conclusion, even though the pathophysiology of muscle injury in the patient herein described has not been clearly established, a possible explanation is that an exaggerated immune response to the ChAdOx1 nCoV-19 vaccine induced severe rhabdomyolysis in a patient with a genetic risk factor. Given the temporal association with the vaccination, monitoring for the possible occurrence of similar cases is recommended.

## Patient Perspectives

Throughout the process, patient and his wife were informed of treatment options and risks. They realized the complexity of the clinical condition and appreciated the multidisciplinary approach.

## Data Availability Statement

The original contributions presented in the study are included in the article/supplementary files. Further inquiries can be directed to the corresponding author.

## Ethics Statement

The studies involving human participants were reviewed and approved by AORN Cardarelli protocol N° 00023621. The patients/participants provided their written informed consent to participate in this study.

## Author Contributions

CE and CP analyzed and interpreted the results, created the figures and tables and actively wrote the manuscript. GF and FB performed the genetic testing evaluation. GG and SF analysed data. LR and SP performed the histological and immunohistochemical evaluation. Critical revision of the manuscript was done by LDN. CE and GA cared for the patient and were actively involved in research investigation. CP took overall responsibility for the research performed in this study and for data integrity. All authors have read and approved the contents of the manuscript and are accountable for all aspects of the work.

## Funding

LDN is supported by the Division of Intramural Research, National Institute of Allergy and Infectious Diseases, National Institutes of Health. CP is recipient of Italian Ministry of Health grant n° RF-2016-02364303 (Ricerca finalizzata).

## Conflict of Interest

The authors declare that the research was conducted in the absence of any commercial or financial relationships that could be construed as a potential conflict of interest.

## Publisher’s Note

All claims expressed in this article are solely those of the authors and do not necessarily represent those of their affiliated organizations, or those of the publisher, the editors and the reviewers. Any product that may be evaluated in this article, or claim that may be made by its manufacturer, is not guaranteed or endorsed by the publisher.
